# Biocompatibility Evaluation of Surface-Modified Orthodontic Wires Using Graphene Layer

**DOI:** 10.3390/ijms26167804

**Published:** 2025-08-13

**Authors:** Joanna Rygas, Maria Szymonowicz, Agnieszka Rusak, Magdalena Wawrzyńska, Piotr Kuropka, Vitalii Boiko, Bartosz Mielan, Dariusz Hreniak, Maciej Dobrzyński

**Affiliations:** 1 Dental Practice, Staszica Str. 46A, 98-300 Wielun, Poland; joanna.rygas@gmail.com; 2 Pre-Clinical Research Centre, Wroclaw Medical University, Bujwida Str. 44, 50-368 Wroclaw, Poland; maria.szymonowicz@umw.edu.pl (M.S.); magdalena.wawrzynska@umw.edu.pl (M.W.); b.mielan@umw.edu.pl (B.M.); 3 Division of Histology and Embryology, Department of Human Morphology and Embryology, Faculty of Medicine, Wroclaw Medical University, Chalubinskiego Str. 6a, 50-368 Wroclaw, Poland; 4 Division of Histology and Embryology, Department of Biostructure and Animal Physiology, Faculty of Veterinary Medicine, Wroclaw University of Environmental and Life Sciences, Norwida Str. 25, 50-375 Wroclaw, Poland; piotr.kuropka@upwr.edu.pl; 5 Institute of Low Temperature and Structure Research, Polish Academy of Sciences, Okolna Str. 2, 50-422 Wrocław, Poland; v.boiko@intibs.pl (V.B.); d.hreniak@intibs.pl (D.H.); 6 Institute of Physics of the National Academy of Science of Ukraine, Prospect Nauky Str. 46, 03028 Kyiv, Ukraine; 7 Department of Pediatric Dentistry and Preclinical Dentistry, Wroclaw Medical University, Krakowska Str. 26, 50-425 Wroclaw, Poland

**Keywords:** graphene, CW-CVD, biocompatibility, cytotoxicity, NiTi archwires stainless steel archwires

## Abstract

The biocompatibility of orthodontic archwires is crucial for ensuring patient safety and the long-term success of orthodontic treatment. This study evaluated the biocompatibility of stainless steel (SS) and nickel–titanium (Ni-Ti) orthodontic archwires, as well as stainless steel metal brackets, before and after the application of a graphene coating. The assessment was based on the materials’ effects on a fibroblast cell line and on the development of a foetal chicken egg embryo. Fibroblasts that had been in temporary contact with steel and NiTi archwires after CW-CVD (cold wall chemical vapour deposition) treatment exhibited changes in morphology in the presence of the material. The materials exhibited moderate cytotoxicity. For metal brackets, the treated samples caused stronger cytotoxic changes in the culture. Unlike graphene-coated implants, where cells were found to directly adhere to the surface, the embryonic tissues did not treat the non-graphene-coated implants as an adhesive material. This study suggests that depositing carbon-based coatings, including graphene, on stainless steel archwires may reduce the cytotoxicity of orthodontic components. Using graphene increases adhesion of the implant surface to membrane-derived cells and the embryonic yolk and does not inhibit the further development of the chicken egg embryo.

## 1. Introduction

The orthodontic arches currently available on the dental market are most often made of stainless steel (SS) or nickel–titanium (Ni-Ti) alloys. The development of nickel–titanium orthodontic arches is one of the most significant advances in orthodontics, introducing innovative solutions to the biomechanics of orthodontic treatment [1]. Nickel–titanium (NiTi) orthodontic archwires offer several advantages over traditional stainless steel (SS) archwires. Thanks to their superelastic properties and shape memory effect, NiTi archwires can exert a constant, gentle force over a long period of time, promoting effective and comfortable tooth movement [2]. Additionally, their lower stiffness (modulus of elasticity) compared to stainless steel enables them to better adapt to the dental arch shape, thereby reducing the risk of soft tissue damage and enhancing patient comfort [3]. Unfortunately, the use of nickel–titanium archwires has some limitations. Using such materials is associated with the deterioration of their mechanical properties over time due to corrosion processes in the oral cavity, as well as toxicity due to the release of nickel ions from their surface [4]. Nickel hypersensitivity, contact dermatitis, erythema, erosive–ulcerative lesions, gingival hyperplasia, and asthma may result from the discharge of nickel ions [5,6,7]. For this reason, various types of protective coatings made of biomaterials are being developed to improve the mechanical properties and biocompatibility of orthodontic archwires and brackets [8,9,10].

One of the biomaterials used to cover orthodontic arches is graphene and its derivatives, such as graphene oxide. Graphene-based nanomaterials represent a technological breakthrough in the development of nanoscale materials [11,12,13]. Graphene and its derivatives have many applications in science and technology thanks to their physical and chemical properties, such as electrical conductivity, transparency, and extreme strength—graphene is 200 times stronger than steel—as well as a large surface-to-volume ratio, chemical stability, unparalleled thermal and electrical conductivity, enhanced cell adhesion and proliferation, and easy synthesis process. Graphene is also low-cost [8,11,14,15,16,17,18,19,20] and has excellent biocompatibility [21,22,23,24,25]. These properties make graphene and graphene-based materials ideal candidates for the surface modification of biological and orthodontic materials [8,16]. One way to modify orthodontic arches is to exploit their chemically inert properties and use their outer surface as a biologically neutral, protective, anti-corrosion film [26,27]. Technologies for depositing graphene coatings on various metal substrates for different applications are well established and primarily based on chemical vapour deposition (CVD) and cold wall chemical vapour deposition (CW-CVD), epitaxy on crystalline substrates, graphite oxide reduction (oxidised graphite) and exfoliation, among other methods [19,28]. This study aimed to determine the biocompatibility of orthodontic appliance components, such as arches and brackets, before and after the application of a graphene layer, by examining their effects on fibroblasts and the development of the foetal hen egg.

## 2. Results

### 2.1. Raman Spectroscopy

**Figure 1:**
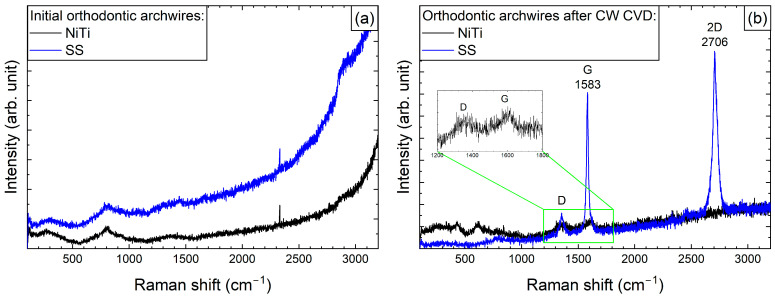
Raman spectra of the initial state (**a**) and after graphene deposition using CW-CVD (**b**) for SS and NiTi orthodontic archwires. The insert shows the D and F modes for the NiTi archwires.

Before depositing graphene, we systematically recorded the Raman spectra of all samples in order to assess any structural changes that occurred during the process. Figure 1a shows the Raman spectra of the initial archwires made of steel and NiTi. Notably, even in the initial samples, we detected a few broad peaks in the range from 200 to 800 cm^−1^, which can be related to α-Fe_2_O_3_ and Fe_3_O_4_ [29]. The similarity in the shape and position of the peaks in the Raman spectra may be due to the presence of a rutile component with Eg and A1g symmetry in the NiTi samples, with positions at 230, 440, and 610 cm^−1^ [30].

After the graphene coating process, Raman spectra were recorded for both arches (Figure 1b). As can be seen in the spectra of the NiTi archwires, two bands are faintly visible, with maxima at 1355 and 1610 cm^−1^, which correspond to the G and D modes of carbon, respectively, as previously described in studies by Pan et al. [10] and Wasyluk et al. [19]. However, the formation of a characteristic 2D band around 2700 cm^−1^, which is a fingerprint of graphene formation, was not observed. Meanwhile, for the steel archwires, bands at 2706 and 1583 cm^−1^ (i.e., the 2D and D modes) were clearly visible, confirming the formation of a high-quality graphene layer, as the G mode at 1355 cm^−1^, responsible for defect formation, had much weaker intensity.

**Figure 2:**
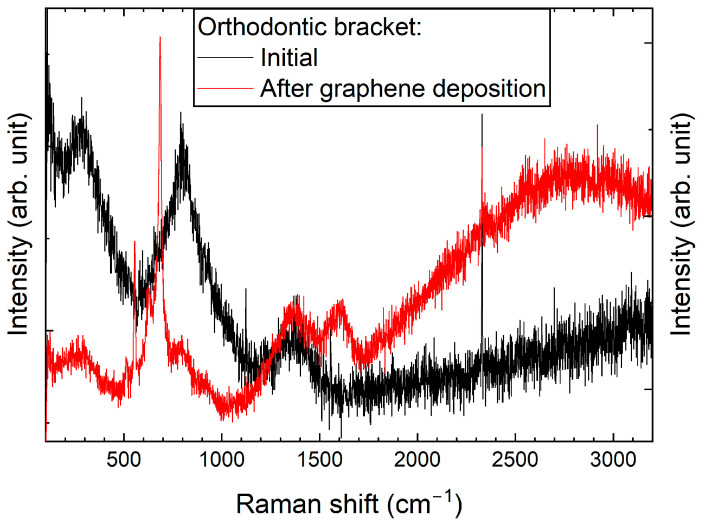
Raman spectra of an orthodontic bracket, taken before and after graphene deposition using CW-CVD.

Similar to the archwires, Raman spectra for brackets were recorded before and after the graphene deposition process (Figure 2). Changes observed within the 100–1000 cm^−1^ range may indicate alterations to the surface structure of the bracket material. Specifically, these changes could suggest the oxidation of elements—primarily metals—that are components of the brackets, or the formation of new metal–carbon composites. Alongside the part of the spectrum where changes related to graphene formation were anticipated, only carbide formation was observed. This may represent an initial step towards developing a graphene layer. Nevertheless, the presence of a graphite-like layer in these samples could significantly enhance their properties.

Significant differences in the formation of graphene on archwires and brackets are evident even when the same deposition procedure is used (see Figure 1 and Figure 2). This variation may be due to differences in their geometrical dimensions, which can result in non-uniform heating at the interface where the archwire contacts the heating element-equipped bracket.

### 2.2. Scanning Electron Microscopy (SEM)

**Figure 3:**
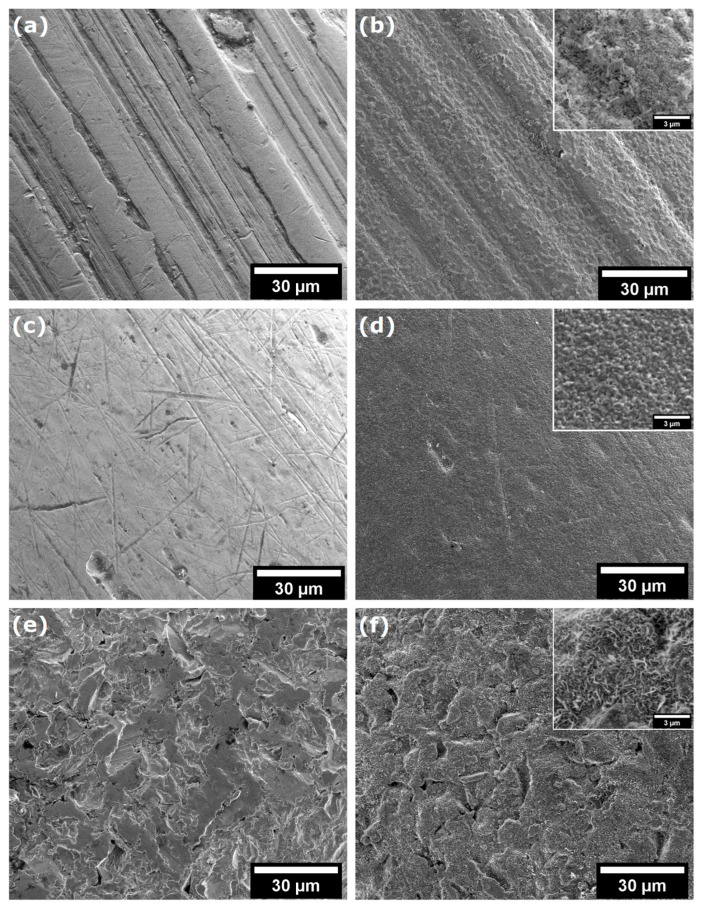
SEM analysis of archwires surfaces: (**a**) initial wires, (**b**) initial wires after CVD, (**c**) initial NiTi, (**d**) initial NiTi after CVD (NiTi-CW-CVD), (**e**) initial brackets, and (**f**) initial brackets after CVD.

As demonstrated in Figure 3a–f, SEM imaging revealed significant variations in the morphological properties of the samples prior to and subsequent to the CVD process. Figure 3a shows the surface of the NiTi archwire, which exhibits typical machining marks such as longitudinal grooves and furrows as well as minor surface defects and microscale roughness. After the CVD process, the NiTi wire is effectively coated with a layer of graphene (see Figure 3b). The presence of graphene, as observed in the SEM image (see inset Figure 3b), is characterised by a fibrous structure with fine-grained ridges, suggesting a high surface texture on a nanometre scale. Figure 3c shows an SEM image on the surface of NiTi wire, which exhibits numerous scratches, microcracks, and surface defects. Furthermore, the observed micropores and inclusions indicate potential heterogeneity in the material structure. The SEM image of NiTi wires after CW-CVD reveals a smooth and homogeneous surface with minimal defects, including micropores and minor impurities (Figure 3d), confirming the high efficiency of the CW-CVD process. The deposited graphene exhibits a fine-grained structure with irregular grains that form a rough texture (see inset Figure 3d). The morphological properties of the brackets before the CVD process are shown in Figure 3e. The surface of this material is characterised by an uneven and rough structure with numerous cracks, holes, and abrasions, which may indicate surface damage. Figure 3f confirms that graphene effectively covered the surface of the brackets. The SEM image (see inset Figure 3f) shows the fibrous structure of graphene, consisting of twisted, irregular fibres that cover it. It is worth noting that the surface of graphene is rough, with small micropores and elevations, which indicates its disordered form.

### 2.3. Biological Studies

#### 2.3.1. Studies of Cytotoxic Effects

The microscopic assessment of the Balb/3T3 mouse fibroblast culture is presented in Table 1. Abnormal changes in the morphology of the Balb/3T3 fibroblasts were observed when they were in contact with the LegendM metal brackets (B) without initial treatment, the Atlas steel arches (D), and the Atlas NiTi arches (G) following the CW-CVD process. These changes were noted in comparison to the control cultures. The changes involved the inhibition of cellular growth, with most cells assuming a spherical shape and undergoing degenerative processes. Cell morphology at the edge of the sample and 1 cm away was normal. The materials exhibited mild cytotoxicity, with a toxicity grade of 2 [31,32,33].

Table 2 presents the morphological changes observed in Balb/3T3 mouse fibroblast cell cultures after 24 h of incubation at 37 ± 1 °C with the tested materials and the control. It also provides a detailed evaluation of these changes based on the presented data and associated toxicity effects.

The mean cytotoxicity score was calculated for each group of tested orthodontic materials. NiTi archwires exhibited a mean toxicity score of 2.5, representing a reduction from 3 (untreated) to 2 (graphene-coated). Similarly, steel archwires exhibited a mean score of 2.5, with the same downward shift after the CW-CVD treatment. The average score for the metal brackets was also 2.5; however, unlike the archwires, the graphene-coated sample showed a higher score [3] than the uncoated one [2]. The highest observed score of 4 was recorded for the graphene deposition fragment (sample F), while the control group showed no cytotoxicity (score 0). These numerical values corroborate the qualitative observations of mild to moderate toxicity and emphasise the variable effects of graphene coating depending on the component type.

#### 2.3.2. In Vivo Microscopic Examination

**Figure 4:**
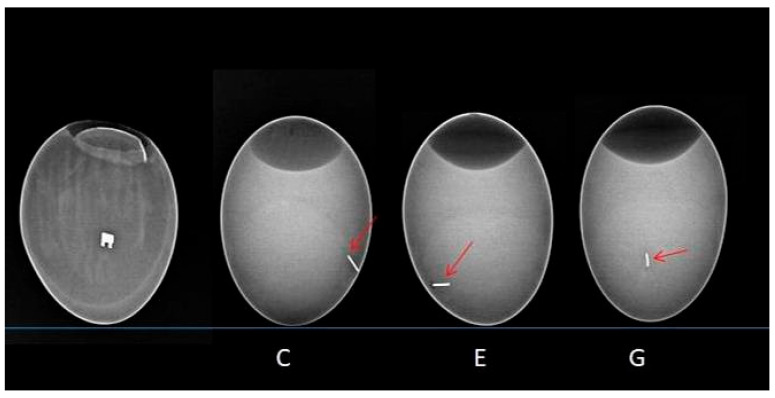
X-ray image illustrating the implanted wires. From left to right are the metal bracket and the archwires from groups C, E, and G. The red arrow indicates the metallic shadow of the archwire.

X-rays of the egg were taken in lateral and oblique planes. In each case, once the implant was inserted into the egg—whether into the yolk or the amniotic membrane—the X-ray images showed that embryonic development was unaffected. The implants did not move. Once all the contents had been removed from the egg, the amniotic fluid and omentum were found to be clear and the correct colour. The metal bracket and archwires in the yolk sac did not induce opacities or disorders related to angiogenesis (see Figure 4).

**Figure 5:**
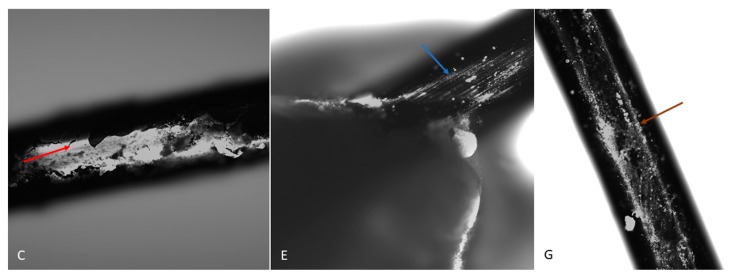
The surface of a dental archwire after incubation in a hen embryo. A visible sheath surrounds the archwire in group C (red arrow). In group E, the archwire surface is visible (blue arrow). In group G, numerous cells are visible directly adjacent to the archwire surface (brown arrow). DAPI. Magnification: 10×.

The microscopic image revealed the formation of a pouch around the metal bracket and archwires. The non-graphene-coated archwires exhibited longitudinal scratches, which were related to the technology used to produce them. As these scratches did not favour cell adhesion, the archwires were surrounded by a membrane composed of yolk-derived material in the form of an unstratified mass of intercellular substance and single cells derived from the gall wall and yolk. Embryonic tissues did not treat the implants in groups C and E (not coated with graphene) as adhesive material; however, in group C, a sheath was produced surrounding the archwires. In contrast, the cells were found to be directly adherent to the surface of the implants in group G (carbon-coated) (Figure 5).

**Figure 6:**
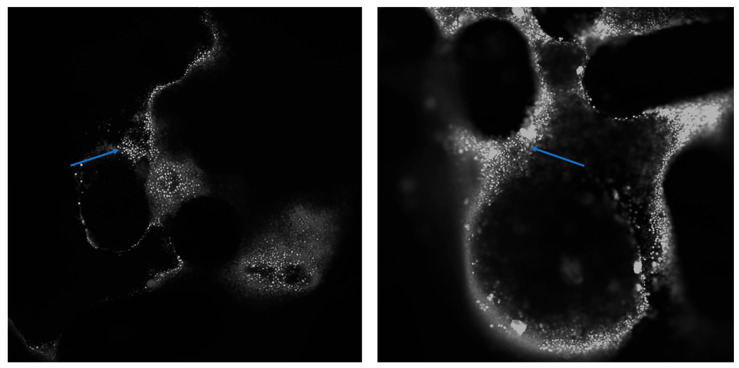
The surface of the bracket after it has been incubated with a chicken embryo. Numerous cells (indicated by the arrow) can be seen on the zipper surface. DAPI. Magnification: 4× (**left**) and 10× (**right**).

This effect was confirmed by confocal microscopy. Additionally, the confocal microscopy revealed cellular elements within the spaces of the metal bracket, forming a pouch that surrounded the bracket (see Figure 6). On the graphene-coated archwires, single cells and newly formed elements of intercellular substance could be seen on the surface. These archwires have a smooth surface and demonstrate adhesion properties for chicken embryo cells.

It is indicated by all that the adhesion of the implant surface to membrane-derived cells and the embryonic yolk is increased by the use of graphene as applied. Consequently, the implant is directly incorporated into the tissue, without a connective tissue capsule forming around it. This mimics the natural process of implant/tissue integration observed, for example, in tendon attachment to bone or tooth tissue fusion (dentine with bone elements of the alveolus).

## 3. Discussion

The formation and diversity of the graphene layers were confirmed by Raman spectroscopy. Spectra of the components were obtained before and after the CW-CVD treatment. A distinct 2D band at ~2700 cm^−1^—a graphene fingerprint—was observed only in the case of the stainless steel archwire (sample D). The other samples, including the NiTi archwire (G), brackets (A), and deposition fragments (F), exhibited disordered carbon structures without clear graphene signatures. This indicates that proper graphene formation occurred primarily on stainless steel. Our study’s confirmation of the graphene coating using Raman spectroscopy is consistent with the approach employed by Wasyluk et al. [19] and Pan et al. [10], who also analysed D, G, and 2D bands to evaluate graphene-based carbon layers deposited via CW-CVD on metallic substrates. Unlike these studies, our work combines surface characterisation with an assessment of the biological response, offering a more comprehensive evaluation of material biocompatibility.

The SEM results clearly showed that CW-CVD changes the microstructure of the materials. Crystalline carbon layers could be observed on all of the materials; however, their microstructure differed depending on the initial surface of the material. This confirms the presence of carbon layers on the materials.

In terms of cytotoxicity, the most significant improvement was observed in sample D, which had a toxicity score of 2, compared to uncoated stainless steel archwire E, which scored 3. This comparison is central to the objectives of the study and confirms that high-quality graphene coatings can reduce cellular toxicity and improve biocompatibility. For NiTi archwires (C vs. G) and brackets (B vs. A), however, the biological improvement was less consistent, likely due to irregular or incomplete carbon deposition. Sample F showed the highest cytotoxicity (score 4), indicating that excessive or poorly controlled deposition can harm cells.

Strong cytotoxic responses in uncoated samples may be associated with the release of metal ions, such as nickel and chromium, which are known to trigger oxidative stress and inflammation [11,21]. These results emphasise the importance of both substrate type and coating quality in determining the biological behaviour of orthodontic materials. The direct contact cytotoxicity test used here is recommended by ISO 10993-5 [31].

This study used the chicken egg model for X-ray and microscopic evaluation of the Atlas NiTi arch and brackets, both before and after the CW CVD process. Using the chicken egg model is a novel and promising method for in vivo studies, providing a reliable and ethically favourable way to evaluate the biocompatibility and cytotoxic properties of biomedical materials [34,35,36]. Numerous studies describe the chicken embryo as an optimal model for various biomedical applications, including the metabolism of chemical compounds. The embryo develops in ovo without a continuous maternal nutrient supply; all essential nutrients (mainly water, lipids, and proteins) are stored in the yolk and gradually transported to the liver, where they are metabolised as required [37,38,39,40]. Fluctuations in hepatic trace elements reflect their mobilisation from yolk stores [37]. The chicken embryo model has been used as an alternative to animal testing in biological, pharmaceutical, and medical research for over a decade [41]. Advantages include accessibility, ease of handling, high bioactivity, and protection from external influences, making it ideal for evaluating both cytotoxicity and the in vivo compatibility of biomaterials [35,36,42,43,44,45]. The rapid development of embryos also allows for the rapid acquisition of results [43,44]. Accordingly, this model has frequently been employed in studies of tumour invasion, angiogenesis, and metastasis [34,46,47,48].

This study found that, following implantation into the yolk sac or amniotic sac, radiographs in lateral and oblique views showed no disruption to embryo development. The implants remained in place. Upon dissection, the amniotic fluid and omentum appeared clear and were the correct colour. No abnormalities in angiogenesis or vitellolysis were observed.

Microscopic examination revealed pouch-like cellular structures surrounding the graphene-coated components. In contrast, uncoated samples with longitudinal surface scratches were enveloped by unstructured material derived from the yolk and composed of the extracellular matrix and isolated cells. These implants were not integrated into the surrounding embryonic tissue. In contrast, graphene-coated implants showed direct cellular adhesion to their smooth surface. Confocal microscopy confirmed this finding by revealing cells and newly formed intercellular material on the surfaces of graphene-coated wires and within bracket spaces.

These results are consistent with previous studies on the biocompatibility of stainless steel and NiTi alloys, which were tested using the direct fibroblast contact method. Studies such as those by Liao et al. (2018) and Tahriri et al. (2019) report that uncoated NiTi exhibits moderate to high cytotoxicity, mainly due to nickel ion release [11,24]. In contrast, the cytotoxicity of stainless steel depends on the surface treatment. In our study, only the graphene-coated stainless steel archwire (D) demonstrated consistent biological benefits.

The biological behaviour of the tested materials is consistent with earlier reports on graphene and carbon-based coatings. For example, Liao et al. (2018) reported that when synthesised correctly, graphene supports cell adhesion and is biocompatible [24]. However, Valentini et al. (2019) and Silveira et al. (2023) demonstrated that disordered or unstable carbon coatings can increase cytotoxicity, especially when thick or poorly controlled [12,25].

Improving cell adhesion and reducing cytotoxicity are essential for achieving clinical success in orthodontic therapy [11,21]. Materials that support cell integration and limit toxic reactions can reduce the risk of inflammation, mucosal irritation, or allergic responses [6,12]. Our results suggest that graphene coatings can enhance cell adhesion and reduce cytotoxicity in vitro, particularly for stainless steel archwires, for which a high-quality graphene layer has been confirmed. However, due to the absence of sample D (a graphene-coated stainless steel archwire) in the in vivo embryo model, these observations cannot be extrapolated to tissue-level interactions. Further in vivo studies including this sample are necessary to confirm its potential for improved implant integration and clinical outcomes.

In clinical conditions, friction between archwires and bracket slots can result in the gradual wear of the graphene coating. While this study did not directly evaluate this, previous research suggests that graphene coatings can reduce friction and wear at the wire–bracket interface due to their excellent lubricating properties [10,28,49]. The graphene layer applied here is only a few nanometres thick and adheres strongly to the metal substrate, especially stainless steel. However, partial abrasion cannot be excluded with prolonged use. According to the current literature, when ingested in small quantities, detached graphene fragments are chemically inert and present low gastrointestinal toxicity [24,25]. Nevertheless, long-term in vivo studies are needed to confirm the durability of the coating and its systemic safety under real oral conditions.

## 4. Materials and Methods

The stainless steel and NiTi archwires (0.016″, natural form) used in this study were obtained from Atlas Orthodontics (Murfreesboro, TN, USA). The metal brackets (Legend, medium-mini, 0.018” slot) were sourced from GC Orthodontics (Breckerfeld, Germany) and are made entirely of medical-grade stainless steel, produced by CNC milling technology (TOMY Inc., Iwaki, Japan). The orthodontic brackets and archwires used in this study are certified, commercially available medical products that have undergone standard biocompatibility testing and have been approved by the manufacturers for market use. Table 3 shows the chemical composition of stainless steel, nickel–titanium archwires, and metal (stainless steel) brackets [49,50,51,52,53,54,55].

Prior to the application of graphene, the arches were cut to a length of 5–10 mm and rinsed in an ultrasonic bath with ethanol. Graphene deposition was performed using a cold wall (CW) reactor with nanoCVD-8G (Moorfield Ltd., London, UK) via chemical vapour deposition (CVD). As the process of graphene film formation depends on the substrate material, parameters were selected for each of the studied materials at which the formation of graphene (or graphite) on the surface of the samples was optimal [56]. Based on optimised CW-CVD deposition parameters and previous reports [19,56,57], the average thickness of the graphene layer was estimated to be approximately 2–5 nm on stainless steel archwires and below 2 nm on NiTi archwires. These values are consistent with those of typical multilayer graphene coatings produced by cold wall CVD. Importantly, such coating thicknesses are significantly below the clinically accepted dimensional tolerance of ±10 µm for orthodontic bracket slots and archwire cross-sections. Therefore, they do not affect the mechanical fit or clinical usability of the components [10,19,28]. Optimal parameters were achieved by controlling the temperatures of the heater, the pressure, and the gas flow rates. The deposition parameters for stainless steel (SS) arches were the same as described in our previous paper and are presented in Table 4 [19]. For NiTi arches, the previously described process was modified and further modified as follows: heating (SP1); annealing in a mixture of argon and hydrogen to remove oxygen residues from the sample surface (SP2); graphene formation and growth (SP3); and cooling down to room temperature and annealing in a mixture of argon and hydrogen (SP4) (Table 4) [57].

### 4.1. Raman Spectroscopy

To confirm the presence and quality of the graphene layer on the orthodontic component, Raman spectra were acquired before and after the deposition process using a Renishaw InVia Raman microscope. These spectra were collected to assess the presence and quality of the graphene layer on the orthodontic archwires following the deposition process. Measurements were taken at 100× magnification using an argon laser with an excitation wavelength of 514 nm and a maximum power of approximately 9 mW at the sample surface, as well as a CCD camera. All measurements were taken under controlled environmental conditions (Renishaw plc, Wotton-under-Edge, Gloucestershire, UK). The detection range for the spectra was 100–3200 cm^−1^.

### 4.2. Scanning Electron Microscopy (SEM)

The samples were analysed using an FE-SEM FEI NovaNano SEM 230 scanning electron microscope (FEI Company, Hillsboro, OR, USA) with an energy dispersive X-ray spectrometer EDAX Genesis XM4. Observations were performed in high vacuum mode at 2500× magnification, using a Wöhler’s electrode voltage of 5.0 kV.

### 4.3. Biological Studies

The in vitro biocompatibility tests included an evaluation of the cytotoxic effects on both a cell line and a chicken egg model. The samples were sterilised in a steam autoclave (134 °C/0.1 atm, Euroklav 23 V-S, MELAG, Berlin, Germany) prior to testing.

The components used in the study were stainless steel and nitinol (NiTi) orthodontic arches, as well as Atlas and Legend steel brackets, all of which are orthodontic appliance components.

#### Cytotoxicity Effect Studies

The following components of the orthodontic appliance were selected for in vitro bioassays:

A/Legend metal brackets (offer CW-CVD), after the CW-CVD process;
B/Legend metal brackets without treatment, initial;
C/Atlas NiTi arches without treatment, initial;
D/Atlas steel arches, after the CW-CVD process;
E/Atlas steel arches untreated, initial;
F/Atlas steel arches, after the CW-CVD process (2 times longer deposition time);
G/Atlas NiTi arches, after the CW-CVD process.

### 4.4. Cell Line

The studies were conducted using the Balb/3T3 mouse fibroblast line (clone A31, American Type Culture Collection, Manassas, VA, USA), which is one of the models used to assess the in vitro cytotoxicity of biomaterials (PN-EN ISO 10993-5:2009) [31]. The Balb/3T3 cells were cultured in Dulbecco’s Modified Eagle’s Medium (DMEM) containing 4.5 g/L of glucose and 25 mM of HEPES (Capricorn, Ebsdorfergrund, Germany), supplemented with 1% L-glutamine, streptomycin, and penicillin (all from Sigma-Aldrich^®^, St. Louis, MO, USA), and 10% calf serum (CS, Sigma-Aldrich^®^), under standard conditions. The incubator was set to 37 °C with a humidity atmosphere and 5% CO_2_ (HERAcell CO_2_ 150i incubator, Thermo Scientific, Waltham, MA, USA).

### 4.5. Cytotoxicity Study—Direct Contact Method

The Balb/3T3 cells were trypsinised using 0.25% Trypsin-EDTA (Sigma-Aldrich^®^), suspended in complete culture medium, and seeded at a density of 1.4 × 10^4^ cells/well in a 48-well plate (TPP, Trasadingen, Switzerland). After 24 h, the material under investigation was introduced to the wells containing the seeded cells. After incubating with the biomaterials for a further 24 h, the morphology of the cells in the vicinity of the disc, within the disc itself, and in the remaining portion of the well was evaluated using an inverted phase-contrast microscope (CKX53, Olympus, Tokyo, Japan) [32,33].

The control group consisted of a fibroblast culture that was maintained in a complete medium under standard conditions and had no contact with the tested material.

The 0–4 cytotoxicity grading scale used in this study was based on ISO 10993-5:2009 guidelines for in vitro direct contact tests. According to this standard, a material is considered cytotoxic if cell viability is reduced by more than 30% compared to the negative control. Scoring was based on quantitative viability assessment (MTT assay) and morphological evaluation of cell layer integrity. The qualitative classification used in Table 1 reflects the ISO thresholds and morphological criteria (e.g., cell lysis, detachment, or rounding) described in the standard [31].

The toxicity gradation is as follows: 0: no changes in cell morphology under the sample; 1: slight degree of reactivity, with single cells degenerating or distorting under the sample; 2: mild, with the zone of changed cells limited to the surface under the material; 3: moderate degree of reactivity, with the zone of changed cells limited to 1 cm around the material; and 4: severe (or strong) degree of reactivity, with the zone of changed cells exceeding the 1 cm limit around the material. A material that has been assessed as having a degree of toxicity of at least the second degree is considered to be cytotoxic [31,32,33].

### 4.6. In Vivo Biological Studies (In Vivo Microscopy)

#### Chicken Egg Model

Based on the obtained cytotoxicity results (see Section 2.3.1), the most extreme results were excluded from the in vivo testing in order to reduce the number of embryos used. Consequently, the number of study groups was reduced to four.

A/metal brackets after CW-CVD process;
C/Atlas NiTi arches without treatment, initial;
G/Atlas NiTi arches, after the CW-CVD process;
E/Steel arch without treatment.

In vivo biological studies assessing the toxicity of the components of the orthodontic appliance were also conducted using a chicken egg model. Prior to the experiment, the eggs were examined using an ovoscope to determine the location of the air chamber and blood vessels. Two holes were made in the shell, taking care not to pierce the subshell membrane. Implants and wires were inserted into the yolk sac through the side wall of the egg, except for the bracket, which was inserted directly into the amnion on day 5. The eggs were then incubated in a HEKA 1 Digital automated incubator (HEKA INKUBATOR Sp. z o.o., Przewoz, Poland) at 37.7 °C with 55% humidity, which was monitored automatically. After the incubation period, the embryos were euthanised by transferring the eggs to a refrigerator at 4 °C for 4 h [58]. Then, an X-ray was taken to locate the implants and perform a macromorphological assessment. After removal from the egg, the implants were fixed in 4% formaldehyde and stained with a mixture of DAPI and propidium iodide for analysis using a Nikon Eclipse 80i fluorescence microscope and a confocal microscope (FV3000, Olympus, Japan).

## 5. Conclusions

In vitro and in vivo studies have demonstrated that applying carbon-based coatings using the CW-CVD method can reduce the cytotoxicity of orthodontic components and improve their biocompatibility. Clear graphene formation was confirmed only on stainless steel archwires among the tested materials, and these exhibited the most significant biological improvement in vitro. Fibroblast cultures exposed to graphene-coated materials exhibited fewer morphological changes and consistently lower cytotoxicity scores compared to untreated controls.

In the chicken embryo model, graphene-coated NiTi archwires and brackets enhanced cell adhesion and tissue integration without impairing embryonic development. However, the most promising material—the graphene-coated stainless steel archwire—was not included in the in vivo evaluation. Consequently, conclusions regarding its in vivo biocompatibility remain preliminary and require further study.

Overall, these findings suggest that high-quality graphene coatings could improve the biological performance of orthodontic appliances, potentially reducing the adverse effects associated with treatment and contributing to better clinical outcomes.

## Figures and Tables

**Table 1:** Morphological images of Balb/3T3 fibroblasts after 24 h of direct contact with the tested material: (a) at the edge of the sample; (b) in the zone up to 1 cm from the sample; and (c) under the sample. Changes in the morphology of Balb/3T3 cells are visible under the tested materials and at the sample edge after CW CVD treatment. Magnification 100×.

Sample	Position a (Edge of the Sample)	Position b (1 cm from Sample)	Position c (Under the Sample)
Bracket (uncoated) [B]	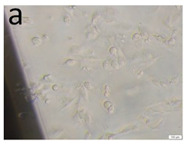	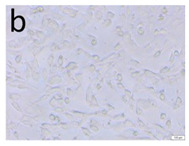	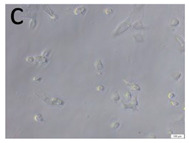
Bracket (carbon-coated) [A]	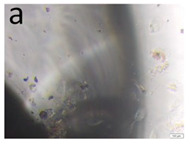	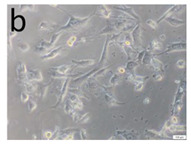	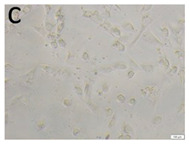
NiTi archwire (uncoated) [C]	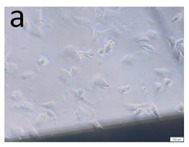	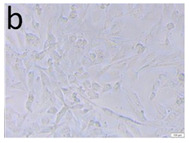	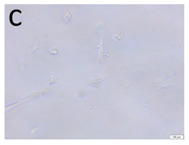
NiTi archwire (carbon-coated) [G]	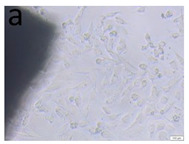	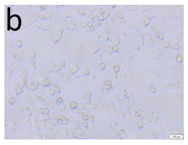	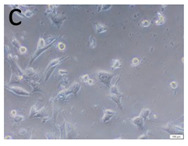
Steel archwire (uncoated) [E]	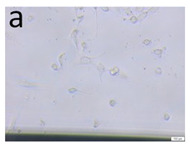	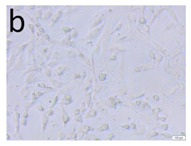	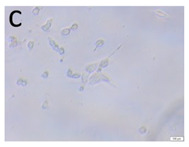
Steel archwire (graphene-coated) [D]	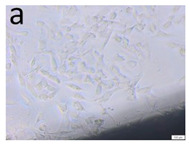	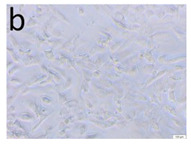	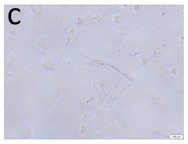
Deposition cut arch [F]	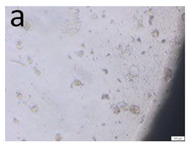	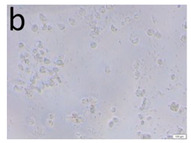	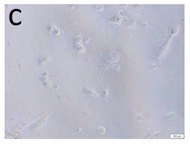

**Table 2:** An evaluation of the material’s cytotoxic activity was performed using the direct contact method. In vitro studies were conducted on the Balb/3T3 fibroblast cells.

Investigated Material	Sample	Morphological Changes in Cell Culture	Evaluation of Changes in Cell Culture	Cytotoxicity
Legend^M^ metal brackets (offer CVD) after CVD process (chemical vapor deposition)	A	Inhibition of cell growth under the tested material and in the area of the sample up to 1 cm; rounded cells with altered morphology were observed	3	moderate
Legend^M^ metal brackets unprocessed, initial	B	Inhibition of cell growth under the tested material; rounded cells with altered morphology were observed	2	mild
Atlas NiTi arches unprocessed, initial	C	Inhibition of cell growth under the tested material and in the area of the sample up to 1 cm; rounded cells with altered morphology were observed	3	moderate
Atlas steel arches, after CW CVD process	D	Inhibition of cell growth under the tested material; rounded cells with altered morphology were observed; normal cells near the sample	2	mild
Atlas steel arches untreated, initial	E	Inhibition of cell growth under the tested material and in the area of the sample up to 1 cm; rounded cells with altered morphology were observed	3	moderate
Arch deposition cut	F	Inhibition of cell growth under the tested material and in the vicinity of the sample up to 1 cm and also at a greater distance from the sample; rounded cells and cell lysis were observed, cell culture destroyed	4	severe
Atlas NiTi arches after CW CVD process	G	Inhibition of cell growth under the tested material; rounded cells with altered morphology were observed; normal cells near the sample;	2	mild
Control (no contact with the tested material)	H	Fibroblast cell shape; no growth inhibition; round proliferative cells in the culture; no altered cell; no lysis	0	no cytotoxicity

**Table 3:** The elemental composition of stainless steel (SS) and nickel–titanium (NiTi) archwires and metal (stainless steel) brackets.

Component Type	Material	Elemental Composition (wt%)	Supplier
Archwire	Stainless steel (AISI 304)	Fe: 68–74%, Ni: 8–12%, Cr: 17–20%, C: 0.08–0.15%	Atlas Orthodontics
Archwire	NiTi (superelastic)	Ni: 50–55%, Ti: 45–50%, Cr: 0–2%	Atlas Orthodontics
Bracket	Stainless steel (medical-grade)	Fe, Cr, Ni, Mo—exact composition not disclosed by the manufacturer; made entirely of stainless steel via CNC milling	GC Orthodontics

**Table 4:** The stages and their corresponding process parameters in the cold wall chemical vapour deposition (CW-CVD) chamber.

	t [s]	T [°C]	P [Torr]	Ar [Sccm]	H_2_ [Sccm]	CH_4_ [Sccm]
stainless steel arches						
SP1	0	90	10	100	20	0
SP2	300	90	10	100	20	0
SP3	0	900	10	100	20	0
SP4	2700	900	10	0	1.2	20
NiTi arches						
SP1	0	1000	0	200	5	0
SP2	3600	1000	0	200	5	0
SP3	150	1000	0	200	5	20
SP4	600	0	0	200	5	0

## Data Availability

Availability of supporting data—the datasets used and/or analysed during the current study are available from the corresponding author on reasonable request.

## References

[1] Brantley W.A. (2020). Evolution, clinical applications, and prospects of nickel-titanium alloys for orthodontic purposes. J. World Fed. Orthod..

[2] Jain A.K., Savana K., Singh S., Brajendu, Roy S., Priya P. (2024). Biomechanical Evaluation of Different Orthodontic Archwire Materials and Their Effect on Tooth Movement Efficiency. J. Pharm. Bioallied Sci..

[3] VS B., Kaul A., Tiwari A., Aliya S., Yadav A., Bera T., Makkad P.K. (2024). Assessment of Various Archwire Materials and Their Impact on Orthodontic Treatment Outcomes. Cureus.

[4] Hussain H.D., Ajith S.D., Goel P. (2016). Nickel release from stainless steel and nickel titanium archwires—An in vitro study. J. Oral Biol. Craniofacial Res..

[5] Wichai W., Anuwongnukroh N., Dechkunakorn S. (2014). Comparison of chemical properties and Ni release of stainless steel and nickel titanium wires. Adv. Mater. Res..

[6] Di Spirito F., Amato A., Di Palo M.P., Ferraro R., Cannatà D., Galdi M., Sacco E., Amato M. (2024). Oral and Extra-Oral Manifestations of Hypersensitivity Reactions in Orthodontics: A Comprehensive Review. J. Funct. Biomater..

[7] Leone F., Gori A., Cinicola B.L., Coletti G., Pignataro E., Martina C., Brindisi G., Anania C., Zicari A.M. (2025). Nickel-induced labial angioedema in a pediatric patient with orthodontic braces: A case report. Ital. J. Pediatr..

[8] Zakrzewski W., Dobrzynski M., Dobrzynski W., Zawadzka-Knefel A., Janecki M., Kurek K., Lubojanski A., Szymonowicz M., Rybak Z., Wiglusz R.J. (2021). Nanomaterials application in orthodontics. Nanomaterials.

[9] Wang P., Luo X., Qin J., Pan Z., Zhou K. (2022). Effect of Graphene Sheets Embedded Carbon Films on the Fretting Wear Behaviors of Orthodontic Archwire–Bracket Contacts. Nanomaterials.

[10] Pan Z., Zhou Q., Wang P., Diao D. (2022). Robust low friction performance of graphene sheets embedded carbon films coated orthodontic stainless steel archwires. Friction.

[11] Tahriri M., Del Monico M., Moghanian A., Tavakkoli Yaraki M., Torres R., Yadegari A., Tayebi L. (2019). Graphene and its derivatives: Opportunities and challenges in dentistry. Mater. Sci. Eng..

[12] Silveira S.R., Sahm B.D., Kreve S., dos Reis A.C. (2023). Osseointegration, antimicrobial capacity and cytotoxicity of implant materials coated with graphene compounds: A systematic review. Jpn. Dent. Sci. Rev..

[13] Powell C., Beall G.W. (2015). Graphene oxide and graphene from low grade coal: Synthesis, characterization and applications. Curr. Opin. Colloid Interface Sci..

[14] Qi X., Jiang F., Zhou M., Zhang W., Jiang X. (2021). Graphene oxide as a promising material in dentistry and tissue regeneration: A review. Smart Mater. Med..

[15] Zare P., Aleemardani M., Seifalian A., Bagher Z., Seifalian A.M. (2021). Graphene oxide: Opportunities and challenges in biomedicine. Nanomaterials.

[16] Lazăr A.I., Aghasoleimani K., Semertsidou A., Vyas J., Roșca A.L., Ficai D., Ficai A. (2023). Graphene-Related Nanomaterials for Biomedical Applications. Nanomaterials.

[17] Saladino M.L., Markowska M., Carmone C., Cancemi P., Alduina R., Presentato A., Scaffaro R., Biały D., Hasiak M., Hreniak D. (2020). Graphene Oxide Carboxymethylcellulose Nanocomposite for Dressing Materials. Materials.

[18] Arkowski J., Obremska M., Kędzierski K., Sławuta A., Wawrzyńska M. (2021). Applications for graphene and its derivatives in medical devices: Current knowledge and future applications. Adv. Clin. Exp. Med..

[19] Wasyluk Ł., Boiko V., Markowska M., Hasiak M., Saladino M.L., Hreniak D., Amati M., Gregoratti L., Zeller P., Biały D. (2021). Graphene coating obtained in a cold-wall CVD process on the Co-Cr Alloy (L-605) for medical applications. Int. J. Mol. Sci..

[20] Rygas J., Matys J., Wawrzyńska M., Szymonowicz M., Dobrzyński M. (2023). The Use of Graphene Oxide in Orthodontics—A Systematic Review. J. Funct. Biomater..

[21] Fahimipour F., Dashtimoghadam E., Rasoulianboroujeni M., Yazdimamaghani M., Khoshroo K. (2018). Collagenous Matrix Supported by A 3D-Printed Scaffold for Osteogenic Differentiation of Dental Pulp Cells. Dent. Mater..

[22] Cobos M., De-La-pinta I., Quindós G., Fernández M.D., Fernández M.J. (2020). Graphene oxide–silver nanoparticle nanohybrids: Synthesis, characterization, and antimicrobial properties. Nanomaterials.

[23] Prakash J., Prema D., Venkataprasanna K.S., Balagangadharan K., Selvamurugan N., Venkatasubbu G.D. (2020). Nanocomposite chitosan film containing graphene oxide/hydroxyapatite/gold for bone tissue engineering. Int. J. Biol. Macromol..

[24] Liao C., Li Y., Tjong S.C. (2018). Graphene nanomaterials: Synthesis, biocompatibility, and cytotoxicity. Int. J. Mol. Sci..

[25] Valentini F., Calcaterra A., Ruggiero V., Pichichero E., Martino A., Iosi F., Bertuccini L., Antonaroli S., Mardente S., Zicari A. (2019). Functionalized Graphene Derivatives: Antibacterial Properties and Cytotoxicity. J. Nanomater..

[26] Zhang R., Han B., Liu X. (2023). Functional Surface Coatings on Orthodontic Appliances: Reviews of Friction Reduction, Antibacterial Properties, and Corrosion Resistance. Int. J. Mol. Sci..

[27] He L., Zhang W., Liu J., Pan Y., Li S., Xie Y. (2024). Applications of nanotechnology in orthodontics: A comprehensive review of tooth movement, antibacterial properties, friction reduction, and corrosion resistance. BioMed. Eng. Online.

[28] Petrone N., Dean C.R., Meric I., Van Der Zande A.M., Huang P.Y., Wang L., Muller D., Shepard K.L., Hone J. (2012). Chemical vapor deposition-derived graphene with electrical performance of exfoliated graphene. Nano Lett..

[29] Kim J.Y., Shin I., Byeon J.W. (2021). Corrosion inhibition of mild steel and 304 stainless steel in 1 m hydrochloric acid solution by tea tree extract and its main constituents. Materials.

[30] Yi Z., Merenda A., Kong L., Radenovic A., Majumder M., Dumée L.F. (2018). Single step synthesis of Schottky-like hybrid graphene—Titania interfaces for efficient photocatalysis. Sci. Rep..

[31] (2009). Biological Evaluation of Medical Devices Part 5: Tests for In Vitro Cytotoxicity.

[32] Tomanik M., Kobielarz M., Filipiak J., Szymonowicz M., Rusak A., Mroczkowska K., Antończak A., Pezowicz C. (2020). Laser texturing as a way of influencing the micromechanical and biological properties of the poly(L-lactide) surface. Materials.

[33] Szymonowicz M., Rybak Z., Fraczek-Szczypta A., Paluch D., Rusak A., Nowicka K., Blazewicz M. (2015). Haemocompatibility and cytotoxic studies of non-metallic composite materials modified with magnetic nano and microparticles. Acta Bioeng Biomech..

[34] Ribatti D., Tamma R. (2019). The chick embryo chorioallantoic membrane as an in vivo experimental model to study multiple myeloma. Enzymes.

[35] Zhang Y., Pham H.M., Tran S.D. (2024). The Chicken Egg: An Advanced Material for Tissue Engineering. Biomolecules.

[36] Sarnella A., Ferrara Y., Terlizzi C., Albanese S., Monti S., Licenziato L., Mancini M. (2024). The Chicken Embryo: An Old but Promising Model for In Vivo Preclinical Research. Biomedicines.

[37] Kuropka P., Dobrzynski M., Tarnowska M., Styczynska M., Dudek K., Leskow A., Wiglusz R.J. (2019). The influence of high doses of a-tocopherol on the content of selected trace elements in the liver of developing chicken embryos in experimentally induced 2,3,7,8-tetrachlorodibenzop-dioxin intoxication. Acta Biochim. Pol..

[38] Ostrowska A., Gostomska-Pampuch K., Leśków A., Kuropka P., Gamian E., Ziółkowski P., Kowalczyk A., Łukaszewicz E., Gamian A., Całkosiński I. (2017). Expression of advanced glycation end-products and NFκB in chick embryos exposed to dioxins and treated with acetylsalicylic acid and α-tocopherol. Poult. Sci..

[39] Gostomska-Pampuch K., Ostrowska A., Kuropka P., Dobrzyński M., Ziółkowski P., Kowalczyk A., Łukaszewicz E., Gamian A., Całkosiński I. (2017). Protective effects of levamisole, acetylsalicylic acid, and α-tocopherol against dioxin toxicity measured as the expression of AhR and COX-2 in a chicken embryo model. Histochem. Cell Biol..

[40] Givisiez P.E.N., Moreira Filho A.L.B., Santos M.R.B., Oliveira H.B., Ferket P.R., Oliveira C.J.B., Malheiros R.D. (2020). Chicken embryo development: Metabolic and morphological basis for in ovo feeding technology. Poult. Sci..

[41] Kucinska M., Murias M., Nowak-Sliwinska P. (2017). Beyond mouse cancer models: Three-dimensional human-relevant in vitro and non-mammalian in vivo models for photodynamic therapy. Mutat. Res. Rev. Mutat. Res..

[42] Nowak-Sliwinska P., Segura T., Iruela-Arispe M.L. (2014). The chicken chorioallantoic membrane model in biology, medicine and bioengineering. Angiogenesi.

[43] Kundeková B., Máčajová M., Meta M., Čavarga I., Bilčík B. (2021). Chorioallantoic membrane models of various avian species: Differences and applications. Biology.

[44] Ribatti D., Annese T., Tamma R. (2020). The use of the chick embryo CAM assay in the study of angiogenic activiy of biomaterials. Microvasc. Res..

[45] Ribatti D. (2023). The chick embryo chorioallantoic membrane patient-derived xenograft (PDX) model. Pathol. Res. Pract..

[46] Augustine R., Alhussain H., Hasan A., Ahmed M.B., Yalcin H.C., Al Moustafa A.E. (2020). A novel in ovo model to study cancer metastasis using chicken embryos and GFP expressing cancer cells. Bosn. J. Basic Med. Sci..

[47] Achkar I.W., Kader S., Dib S.S., Junejo K., Al-Bader S.B., Hayat S., Bhagwat A.M., Rousset X., Wang Y., Viallet J. (2020). Metabolic signatures of tumor responses to doxorubicin elucidated by metabolic profiling in ovo. Metabolites.

[48] Rezzola S., Loda A., Corsini M., Semeraro F., Annese T., Presta M., Ribatti D. (2020). Angiogenesis-Inflammation Cross Talk in Diabetic Retinopathy: Novel Insights From the Chick Embryo Chorioallantoic Membrane/Human Vitreous Platform. Front. Immunol..

[49] Al-Horini O., Hajeer M.Y., Baba F. (2022). Evaluating the Elemental Composition, Transformation Behavior, Crystalline Structure, and Mechanical Properties of Three 0.016-Inch by 0.022-Inch Nickel-Titanium Archwires: An In Vitro Study. Cureus.

[50] Grygiel D., Hoppe V., Zięty A., Rutkowska-Gorczyca M. (2018). Evaluation of the differentiation of structural and physicochemical properties of orthodontic wires of AISI 304 stainless steel. Eng. Biomater..

[51] Brüngger D., Koutsoukis T., Al Jabbari Y.S., Hersberger-Zurfluh M., Zinelis S., Eliades T. (2019). A comparison of the compositional, microstructural, and mechanical characteristics of Ni-free and conventional stainless steel orthodontic wires. Materials.

[52] Uysal I., Yilmaz B., Atilla A.O., Evis Z. (2022). Nickel titanium alloys as orthodontic archwires: A narrative review. Eng. Sci. Technol. Int. J..

[53] Kuc A.E., Kotuła J., Nawrocki J., Dobrzyński M., Wiglusz R.J., Watras A., Sarul M., Lis J., Kawala B. (2024). Properties and Application of the Gummetal Wire for the Treatment of an Open Bite—Brief Narrative Review and Case Report. Appl. Sci..

[54] Orthodontics G.C. (2021). Global Experts in Orthodontics: Product Catalog. https://oneorthodontics.com/catalogos2021/GCOA_Catalog_04_Complete_04122021s.pdf.

[55] Orthodontics G.C., GmbH E. (2025). GC Orthodontics Europe GmbH Official Website. https://www.gc.dental/ortho/en/products/legend.

[56] Chen X., Zhang L., Chen S. (2015). Large area CVD growth of graphene. Synth. Met..

[57] Katona B., Bognár E., Berta B., Nagy P., Hirschberg K. (2013). Chemical etching of nitinol stents. Acta Bioeng. Biomech..

[58] Aleksandrowicz E., Herr I. (2015). Ethical Euthanasia and Short-Term Anesthesia of the Chick Embryo. Altern. Anim. Exp..

